# The New Concept of Physiological “Riolan’s Arch” and the Reconstruction Mechanism of Pathological Riolan’s Arch After High Ligation of the Inferior Mesenteric Artery by CT Angiography-Based Small Vessel Imaging

**DOI:** 10.3389/fphys.2021.641290

**Published:** 2021-06-22

**Authors:** Ying Wang, Weibin Shu, Aimie Ouyang, Lei Wang, Yuping Sun, Guoqin Liu

**Affiliations:** ^1^Department of Radiology, Jinan Central Hospital Affiliated to Shandong First Medical University, Jinan, China; ^2^Tumor Research and Therapy Center, Shandong Provincial Hospital Affiliated to Shandong First Medical University, Jinan, China; ^3^Department of Gastroenterology, Jinan Central Hospital Affiliated to Shandong First Medical University, Jinan, China; ^4^Department of Oncology, Jinan Central Hospital Affiliated to Shandong First Medical University, Jinan, China

**Keywords:** middle colic artery, inferior mesenteric artery, left colic artery, Riolan arch, colorectal cancer

## Abstract

**Background:**

Due to the complexity of anatomical relationship between superior mesenteric artery (SMA) and left colic artery (LCA), there is no unified anatomical concept of “Riolan’s arch.” There is no consensus as to tie off the inferior mesenteric artery (IMA) at its origin or just below the origin of the left colic artery during radical surgery of sigmoid colon and rectal cancers. The aim of the study is to investigate the anatomy of shortcut anastomotic branches (adjacent branches) of SMA at splenic flexure and to explore how the shortcut pathway (Riolan’s arch) was formed, as the compensation of anastomotic branches between MCA and LCA under pathological conditions and the reconstruction and the mechanism of pathological Riolan’s arch after high ligation of the inferior mesenteric artery.

**Methods:**

Between January 2018 and May 2020, patients with colorectal cancer who underwent CTA before surgery were enrolled in the study. The anatomy of shortcut anastomotic branch of SMA and LCA was investigated by volume rendering technique (VR) and maximum-intensity projection (MIP). GE’s small vessel extraction technology (selected VR) was used to directly display these shortcut anastomotic branches on a map and to establish their three-dimensional anatomical classification. Then, we used the axonometric drawing to make the model more exact. Next, combining with some cases of pathological Riolan’s arch and basing on hydrodynamic principle, we speculate the mechanism of collateral circulation. Finally, based on the retrospective study of high ligation cases and combined principles of fluid mechanics, we show how these shortcut anastomotic branches evolved into Riolan’s arch.

**Results:**

We report the classification of the ascending branch of LCA (which approaches the splenic flexure) and the left branch of MCA, display these shortcut anastomotic branches on a map, and establish their three-dimensional anatomical classification. We found that Riolan’s arch is a shortcut pathway for the compensation of anastomotic branches, between MCA and LCA under pathological conditions, and that the formation mechanism of shortcut path accords with the principle of hydrodynamics.

**Conclusions:**

Our results show the mechanism of pathological Riolan’s arch formation and provide new anatomic thinking for the battle between high and low ligation of IMA in colorectal cancer surgery.

## Introduction

In recent years, laparoscopic surgery has been widely used in the management of colorectal cancer. However, no clear consensus has been established, as to whether it is most appropriate to tie off the inferior mesenteric artery (IMA) at its origin (high ligation) or just below the origin of the left colic artery (LCA) (low ligation) during radical surgery of sigmoid colon and rectal cancers (Chin et al., 2008; Titu et al., 2008; Yasuda et al., 2016; Cirocchi et al., 2019; Miskovic et al., 2019).

Many surgeons prefer to perform high ligation of the inferior mesenteric artery (IMA) during rectal cancer surgery, in order to accomplish a total lymphadenectomy and better mesenteric mobilization (Kanemitsu et al., 2006; Chin et al., 2008). Other surgeons recommend low ligation of the IMA, in order to protect the blood supply of the anastomosis. Insufficient blood supply in the anastomosis is considered a risk factor, for postoperative anastomotic leakage in patients undergoing colorectal cancer surgery. However, high ligation can ensure blood supply in most cases. There are some controversial theories that attempt to offer explanations for this, including theories regarding “Riolan’s arch” and the artery of Drummond (Titu et al., 2008).

These arterial patterns were established previously by means of postmortem angiography or digital subtraction angiography (DSA) (Griffiths, 1956; Yada et al., 1997). However, these methods do not provide an accurate reflection of the arterial distribution pattern and the relationship of each branch of the main colon artery in the physiological state (analogous to a parallel circuit). In particular, they cannot reflect the compensatory mechanism (reconstruction of the parallel circuit to form Riolan’s arch after high ligation of IMA) and blood flow direction under pathological conditions. In short, those methods do not account for the compensatory reactions that occur when the primary arterial pathways have been blocked for surgery; these compensatory reactions may cause the creation of pathological Riolan’s arch.

Riolan’s arch’s existence has been debated for years. It is believed to be an important anastomotic artery between the left branch of the middle colic artery (MCA) from the SMA and the ascending LCA branch from the IMA (Tommaseo, 1958; Cheng and Gao, 1995). It is believed to play an important role in providing blood supply to the proximal intestine, near the origin of the anastomosis. However, there are still many doubts regarding the existence of Riolan’s arch, and it remains a controversial topic (Lange et al., 2007). Some experts described Riolan’s arch as a hypertrophied and tortuous marginal artery present in chronic atherosclerotic pathologies of the mesenteric vasculature (Bruzzi et al., 2019). There is still no clear anatomical explanation for the formation of Riolan’s arch in the physiological state. What are the conditions that could lead to Riolan’s arch or “potential Riolan’s arch?”

Through computed tomography angiography (CTA) of the IMA, we observed some Riolan’s arch under pathological conditions and plan to elucidate the formation of Riolan’s arch under physiological conditions. We reviewed the literature surrounding Griffiths’ research (Meyers, 1976), wherein he reported that “The left colic artery as it approaches the splenic flexure usually divides about two inches from the gut. One branch passes to the distal loop of the flexure, the other branch goes to the transverse colon, thus affecting an anastomosis with the middle colic’s artery’s left branch, where the terminal form of the left colic artery should always be preserved after ligation of the inferior mesenteric at its origin, since it forms a second marginal artery, otherwise the distal colon is dependent solely on the very fine marginal artery of the splenic flexure” (Meyers, 1976). This description strongly indicated that the terminal branch is one of the most important part of Riolan’s arch. The anatomical morphology of the “terminal” of the left branch of the MCA also needs further investigation. The middle colic artery has been described as “the main supply to the splenic flexure in about one-third of individuals,” but this does not provide an explanation for the other two-thirds of individuals. Furthermore, due to the special location of the transverse mesocolon, it is difficult to display the blood supply surrounding it. Therefore, we observed the physiological pattern of these blood vessels.

Because the anastomosis of the splenic flexure is formed in three-dimensional space, we can only investigate and display this relationship accurately using a three-dimensional model. All previous research methods (such as DSA) consist of two-dimensional modeling, and the branch construct of the SMA and IMA could not be investigated at the same time. In addition, the branches at the splenic flexure are grades 2 and 3 branches of the main artery supply, and only three-dimensional small-vessel imaging technology can track this clearly.

Therefore, we sought to investigate the small vessels through adjusting the shaft position angle of maximum-intensity projection (MIP) and selective VR (small-vessel extraction technology), then chose a three-dimensional perspective angle to demonstrate their relationship. What is more, previous studies did not address the principle of pathological Riolan’s arch formation. Through the study, we found that the formation mechanism of blood flow pathway conforms to the principle of fluid mechanics.

In this report, we try to investigate the existence form of the “physiological Riolan’s arch” by using VR and adjusted-angle MIP. We summarize six types of shortcut anastomotic branches. Retrospective study of 57 cases of the actual blood flow path after high ligation of IMA also supports the hypothesis that the shortcut pathway was formed by the compensation of the shortcut anastomotic branches, following the principle of fluid mechanics, and the two-way blood supply to the far side together with the limbic arch. All of them followed the principle of fluid mechanics.

Finally, we use small blood vessel extraction technology to display the three-dimensional anatomical classification of the shortcut anastomotic branches and show the whole blood flow path after high ligation of the inferior mesenteric artery. These studies provide an anatomical thinking for part settling dispute between high ligation and low ligation of the IMA in rectal surgery.

## Materials and Methods

### Patient Selection

In this study, we reviewed a total of 300 patients with colorectal cancer who underwent routine abdominal and pelvic enhancement CT before surgery at Jinan Central Hospital from January 2018 to March 2020. The scan data of arterial phase were reconstructed into 1.0 mm thin-layer image data. There were 57 cases of routine reexamination after IMA high ligation from March 2019 to March 2020 that were used for further analysis. The 1.0-mm CT arterial phase thin-slice image data were used for CTA reconstruction and analysis. Because this study was both of basic research in cross discipline and retrospective study, information was provided to the participants in place of informed consent, and they were given the right to opt out. This study was approved by the Ethics Committee of Jinan Central Hospital.

### Three-Dimensional CT Angiography Protocol

The 64-slice multidetector-row computed tomography (MDCT) scanner generates 0.75 mm image slices that can be reconstructed into 1.0 mm image data. Image processing analysis was performed using a 3D volume rendering (VR) technique with the Extended Brilliance Workspace (EBW) software. The MIP images were evaluated using VITAL Image Management System (VIMS, Vitrea^®^ 2). The selected VR image (based on small-vessel extraction technology) was evaluated using AW VolumeShare 5, GE Medical Systems.

### Classification

In accordance with the method of Murono et al. (2015), we utilized CT angiography to fully visualize the left branch of the middle colic artery and the grades 2 and 3 branches of the ascending left colic artery at the splenic flexure in 267 of the 300 patients (in some cases, the scanning was done earlier and the structure was unclear). All patients met the inclusion criteria (through VR technology, the main branch are clear), and the final study group had a median age of 55.6 years (range, 25–75 years).

**FIGURE 1:**
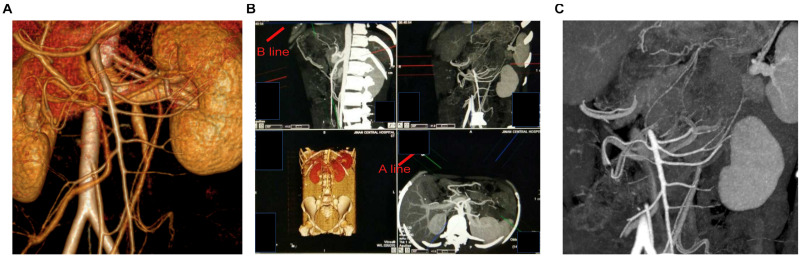
**(A)** Image processing analysis was performed using a 3D volume rendering (VR) technique with the extended brilliance workspace (EBW). **(B)** By adjusting the A-line and B-line, we can get the best perspective. **(C)** The maximum-intensity projection (MIP) images were evaluated using VITAL Image Management System.

**FIGURE 2:**
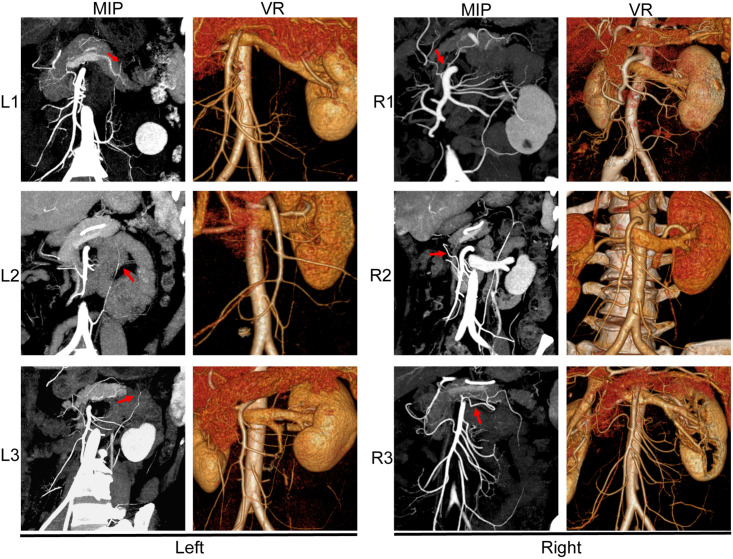
The anatomical patterns of the LCA **(left)** and MCA **(right)**.

The distribution of blood vessels was further detected using VR and MIP (Sparacia et al., 2007) in 130 (out of 172) cases. By adjusting the angle of the A or B axis (Figure 1), we could visualize the left branch of the middle colic artery and the grades 2 and 3 branches of the ascending left colic artery at the splenic flexure in one picture. When the accessory middle colic artery (AMCA) travels over the lower edge of the pancreas, it can be imaged simultaneously with the branch of the IMA within a certain thickness. Combined with VR, we can accurately observe the left branch of the MCA (Figure 2).

**FIGURE 3:**
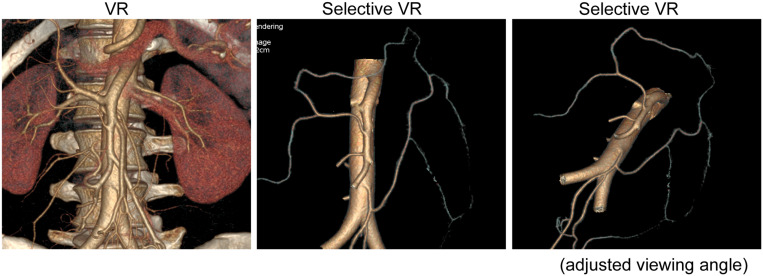
The GE’s small vessel extraction technology was used to display the classification and anastomosis of shortcut branches intuitively. We adjusted the viewing angle to the axonometric drawing angle.

**FIGURE 4:**
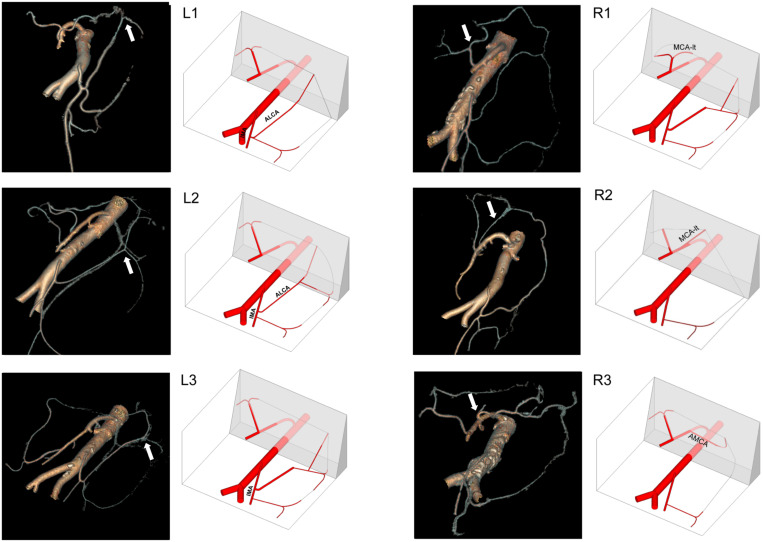
Three-dimensional anatomical classification of the shortcut branch of the LCA **(left)** and MCA **(right)**.

Then, GE’s small-vessel extraction technology (selected VR) was used to directly display these shortcut anastomotic branches on a map and to establish their three-dimensional anatomical classification. We then used the axonometric drawing to make the model more exact. During the imaging process, we found a specific perspective angle that can better display the three-dimensional relationship of each anastomotic branch (Figure 3) and made a three-dimensional structural model map revealing the classification of shortcut anastomotic branches (Figure 4).

**FIGURE 5:**
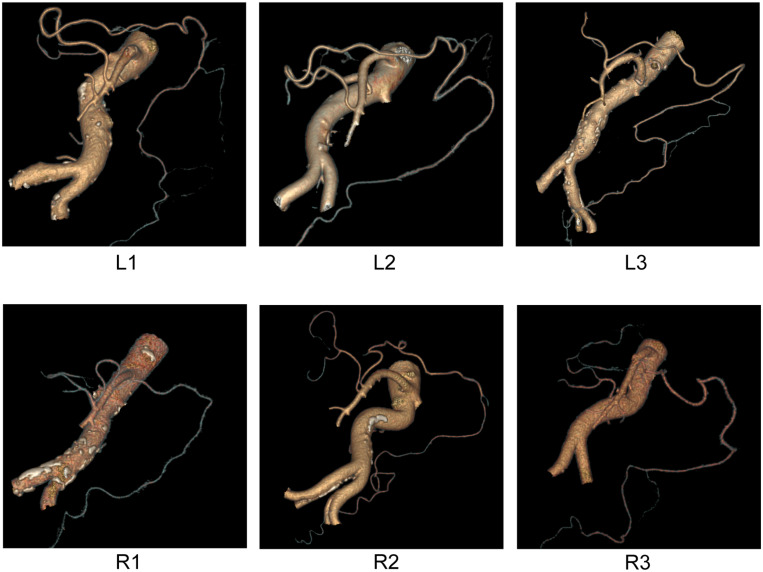
The blood flow pathway after high ligation. The collateral circulation (compensatory pathway) evolved from the shortcut anastomotic branches.

Finally, we investigated the blood flow pathways of 57 high ligation cases and found that the collateral circulation (compensatory pathway) evolved from the shortcut anastomotic branches (Figure 5).

## Results

### Branching Pattern of the Ascending Branch of Left Colic Artery (Which Approaches the Splenic Flexure)

Most MIP images and almost all GE selective VR images can show the descending colon and the limbic arch. Through adjusting the angle, we can directly show that the ascending branch of LCA and left branch of MCA are anastomosed in the splenic flexure, which also proves that we have been able to image all the three to four branches of each blood vessel. The ascending branch of the LCA travels to the left colic angle in order to constitute the left pillar of the marginal artery.

As reviewed by Bruzzi et al. (2019), the terminal bifurcation of the ascending branch of the LCA into a “’V” shape represents a functional and valid anastomotic arterial arch that supplements the marginal artery. As shown in Figure 2, we found three branching patterns of the ascending branch of the LCA: L1 (10.5%), L2 (13.9%), and L3 (24.3%) (Table 1). In L1, the ascending branch of the LCA travels to the medial side of the left kidney hilum and reaches the left 1/3 mesocolon of the transverse colon directly, without branches to descending colon. In L2, the ascending branch of the LCA divides into two branches (left and right) and terminates into a V shape at the level of the renal hilum; one branch travels upward to the transverse colon, and the other branch extends to the upper part of the descending colon. In L3, the ascending branch of the LCA turns left at the renal hilum toward the splenic flexure, then divides into two branches and terminates into a small V shape.

**TABLE 1:** Distribution of the ascending branch of LCA.

	Type	Number	Percentage (%)
LCA (total 267)	L1	28	10.5
	L2	37	13.9
	L3	65	24.3
	None	137	51.4

### Branching Pattern of the Left Branch of MCA and the AMCA

Due to the limitation of techniques and the diverse locations of the transverse colon’s left mesocolon, the main splenic artery of the transverse colon has not been clearly reported.

Here we classified the left branching pattern of the MCA and the AMCA (Ito et al., 2019) into three types: type R1 (10.1%), type R2 (14.5%), and type R3 (32.6%) (Table 2). The type R1 branching pattern is characterized by the right and left branches of the MCA, originating from the short common trunk, to supply the left transverse colon. In type R2 (14.6%), the right and left branches of the MCA originate separately from different origins of the SMA; the left branch of the MCA originates higher from the SMA and directly distributes to the mesocolon. In type R3 (32.6%), the AMCA originates from the SMA at the inferior border of the pancreas and supplies the transverse colon at the splenic flexure. The AMCA of type R3 also occasionally originates from the celiac trunk (Figure 2).

**TABLE 2:** Distribution of the left branch of the MCA and AMCA.

	Type	Number	Percentage (%)
The left branch of the MCA and AMCA (total 267)	R1	27	10.1
	R2	39	14.6
	R3	87	32.6
	None	114	42.7

### Shortcut Anastomotic Branches Three-Dimensional Distribution

In order to better display the shortcut anastomotic branches, we used GE’s small-vessel extraction technology to display the classification and distribution of shortcut branches intuitively (Figure 4). We sought assistance from a structural engineer to construct the three-dimensional anatomical classification of the shortcut branches (Figure 3) out of the axonometric drawing. We can observe an increasing number of thin blood vessels and the winding of blood vessels more intuitively. In particular, we find the shortcut branches of both directions shake hands at marginal arterial arch.

### Formation and Mechanism of a Compensatory Pathway After High Ligation of the IMA

We investigated the blood flow pathways of 57 high ligation cases and found that the collateral circulation (compensatory pathway) evolved from the shortcut anastomotic branches: The left branch of MCA and anastomotic site dilated and blood flow increased, anastomotic branch of ascending branch of LCA dilated, and reverse blood flow occurred. The formation mechanism follows the fluid mechanics. Therefore, the second blood supply to the distal intestine is combined with the marginal arch. Of note, the occurrence rate of the accessory colon middle artery is higher (Figure 5).

## Discussion

For gastrointestinal surgeons, an operation’s success hinges on a full preoperative understanding of the blood supply of the splenic flexure. The splenic flexure is the junction of the transverse and descending colon; unfortunately, its blood supply has rarely been investigated. The number and exact location of the colic arterial arches that connect the superior and inferior mesenteric arteries at the splenic flexure is largely unknown (Fukuoka et al., 2017). This connection of the two arterial systems is essential in colorectal surgeries involving ligature of the inferior mesenteric artery at its origin (especially in cancer patients), because it allows for vascularization of the descending colon by the SMA.

Previous studies yielded contradictory results mainly due to limitations in methodology. Analysis and interpretation of two-dimensional images cause some misunderstandings that can only be corrected with three-dimensional modeling. In recent years, multidetector-row computed tomography (MDTC) and three-dimensional computed tomography angiography (3D-CTA) have become frequently used for the evaluation of vascular anatomy.

**FIGURE 6:**
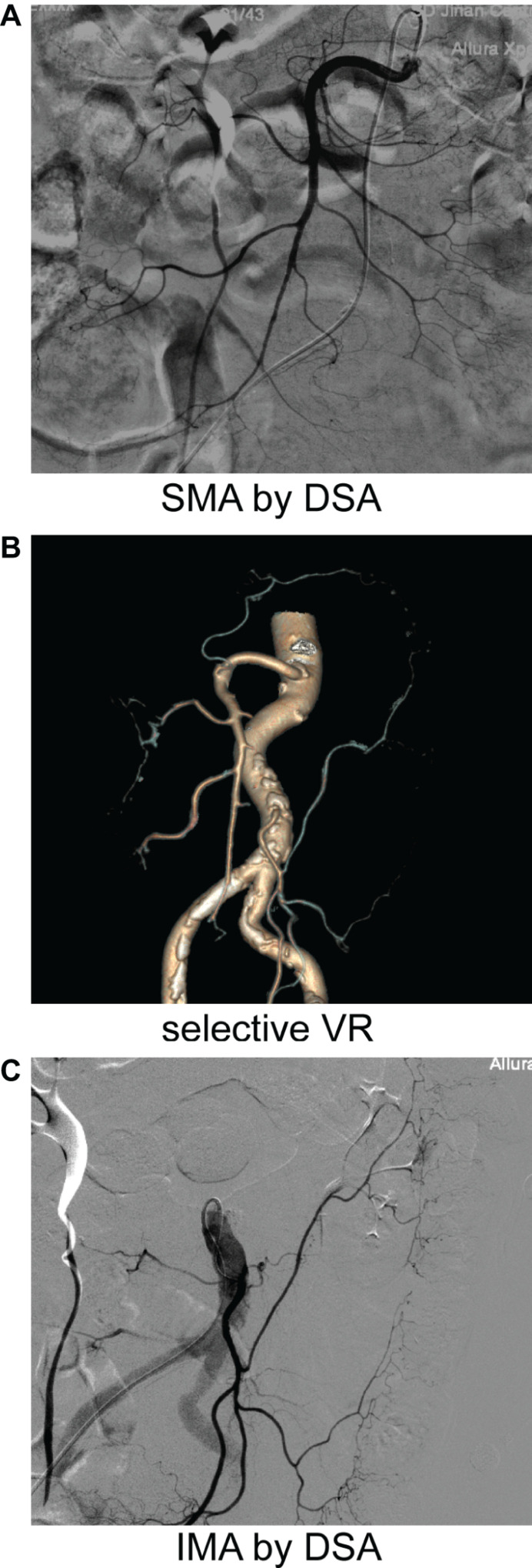
DSA and CTA images from the same patient separately. The DSA was taken **(A**,**C)** after enhanced CT **(B)** in the same patient.

In this study, we evaluated the vascular anatomy of the splenic flexure in a large number of patients, using 3D-CTA to get a three-dimensional perspective. The selected VR technology enables us to show small and curved blood vessels and provides evidence of shortcut anastomosis between the MCA and the LCA. As shown in Figure 6, DSA technology can display the SMA (Figure 6A) and IMA (Figure 6C) separately, but it is unable to demonstrate the relationship between them. By using selective VR technology, we can display the SMA and the IMA on a single map simultaneously, which allows us to find their connection.

Our results show how the left branch of the MCA distributes to the left 1/3 of the mesocolon. This is very important, because in rectal cancer surgery, this left branch serves as a compensatory pathway for upstream blood supply.

The AMCA is a branch artery originating directly from the SMA and is more proximal than the first jejunal artery. It has recently been identified preoperatively, because of recent advances in imaging technologies. The role of the AMCA in colon surgery is very important, but there is little known about its branching pattern.

In the past, the anatomical structure of the MCA was mainly obtained by DSA, which demonstrates the projection of blood vessels but loses information by simplifying three-dimensional space into a two-dimensional model. The depth of the angiography catheter entering the SMA may affect the appearance of the branch vessels that originate higher than the SMA. Therefore, DSA cannot provide the exact location of blood vessels. Without a precise reference system, it is difficult to determine a blood supply map of the upper SMA branches, and some vessels could not be named accurately.

Our method accurately identified the AMCA and demonstrated that in some patient the left branch point of the MCA originated higher and separately from different origins of the SMA (not from a common trunk) (as shown in Figure 6).

Current knowledge cannot explain the conjunction of the left MCA branch and the ascending LCA branch. Riolan’s arch is believed to be an important anastomotic artery between the left MCA branch from the SMA and the ascending LCA branch from the IMA. Riolan’s arch ensures blood supply reaches the anastomotic opening’s proximal intestine after high ligation in rectal surgery. If Riolan’s arch is missing, there is an increased risk of insufficient blood supply. However, there are many doubts regarding the existence of Riolan’s arch (Lange et al., 2007). The Riolan’s arch was determined to represent a hypertrophied and tortuous marginal artery, present during chronic atherosclerotic pathologies of the mesenteric vasculature (Bruzzi et al., 2019). In what form does Riolan’s arch exist in the physiological state? There is no explicit report.

We found here that the proportion of Riolan’s arch in the physiological state was much higher than that in the classical reported state (about 10%), and there was no “potential blood vessel.” It has been previously established that the blood supply of the colon is divided into different sections (the main arteries of each section are similar to the parallel circuit, which is well shown in both DSA and selective VR). When these secondary and tertiary branch vessels extend into the marginal arch, they are also the main arteries of the corresponding colon under physiological conditions.

In this experiment, we sought to explain why the collateral circulation, Riolan’s arch, is formed by compensation of shortcut anastomotic branches. This can be explained by the original principle of fluid mechanics (Jiang et al., 2013). As shown in Figure 6, with the numbers of branches on marginal arterial arch, the total diameter of parallel vessels also increases while the blood flow gradually slows down, achieving a dynamic balance at the point of anastomosis. When the main artery of one side is occluded or ligated, the fluid pressure of that side disappears. This results in increased blood flow to the opposite side due to the redistribution of the proximal blood, so the reverse blood flow will be maintained through the original blood vessels of the affected side, in order to preserve the intestinal blood flow. We observed the establishment of collateral circulation in 57 cases after high ligation of the IMA, and the results support our hypothesis.

We took advantage of selective VR, which can be used to track the blood flow of axial sections. It is similar to tracking the blood flow of an angiography, so as to find the marginal anastomosis. Selective VR can accomplish simultaneous imaging of the SMA and the IMA and can rotate at any angle during the mapping process. Thus, it is able to accurately confirm the starting point and arrival position of each vessel. We were able to conclude that a high proportion of AMCA and L1 and L2 correlates with classic central Riolan’s arch.

The shortcut anastomotic branches all connect at the marginal arch, when the superior branch is occluded or ligated. As long as the exit point of the anastomotic branch is not broken, the anastomotic branch compensates by expanding and the shortcut branch shows retrograde blood flow. The inside path is a shortcut relative to the marginal arch, which is the so-called Riolan’s arch.

The efficiency of the shortcut path is stronger than that of the marginal arch. Before the shortcut path reaches the marginal arch, there is no branch and minimal change in blood flow. The marginal arch has many small branches, and the total vessel diameter keeps increasing, resulting in a continuous decrease in blood flow. After a certain distance, the blood flow is very slow and the blood supply efficiency is low.

These six shortcut routes can provide an anatomical basis for ending the controversy between high and low ligations. In order to ensure the blood supply of the proximal colon of the anastomotic opening, high or low ligation should each be considered based on the circumstances. When there is only L1 or no right anastomotic branch, the best method is to perform a low ligation.

The shortcut branch becomes an important vascular pathway under certain circumstances. Our discovery is helpful to international surgeons. Oriental doctors tend to maintain a longer sigmoid colon in the operation, which means there should be twice as many channels to supply blood to the distal colon, instead of relying on the marginal arch alone.

Western doctors tend to keep only the descending colon, but they need to disconnect the inferior mesenteric vein at a high level in order to free the splenic curve and extend the proximal colon of the anastomosis. Therefore, they need to pay attention to the relationship between the inferior mesenteric vein and the shortcut pathway. For example, in the case of the middle accessory colon artery, they should disconnect the lower position of the inferior mesenteric vein.

## Data Availability Statement

The raw data supporting the conclusions of this article will be made available by the authors, without undue reservation.

## Ethics Statement

The studies involving human participants were reviewed and approved by the Human Ethics Committee of Jinan Central Hospital Affiliated to Shandong University. The patients/participants provided their written informed consent to participate in this study. Written informed consent was obtained from the individual(s) for the publication of any potentially identifiable images or data included in this article.

## Author Contributions

GL designed the study. YW and WS analyzed the data and drafted the manuscript. AO participated in the design of the study and helped draft the manuscript. LW performed the statistical analysis. YS helped interpret the data. All authors read and approved the final manuscript.

## Conflict of Interest

The authors declare that the research was conducted in the absence of any commercial or financial relationships that could be construed as a potential conflict of interest.
